# Role of the Perigenual Anterior Cingulate and Orbitofrontal Cortex in Contingency Learning in the Marmoset

**DOI:** 10.1093/cercor/bhw067

**Published:** 2016-04-29

**Authors:** Stacey A. W. Jackson, Nicole K. Horst, Andrew Pears, Trevor W. Robbins, Angela C. Roberts

**Affiliations:** 1 Department of Psychology; 2 Behavioural and Clinical Neuroscience Institute, University of Cambridge, Cambridge CB2 3EB, UK; 3 Department of Physiology, Development and Neuroscience, University of Cambridge, Cambridge CB2 3DY, UK

**Keywords:** goal-directed action, habit, obsessive-compulsive disorder, prefrontal, primate

## Abstract

Two learning mechanisms contribute to decision-making: goal-directed actions and the “habit” system, by which action-outcome and stimulus-response associations are formed, respectively. Rodent lesion studies and human neuroimaging have implicated both the medial prefrontal cortex (mPFC) and the orbitofrontal cortex (OFC) in the neural basis of contingency learning, a critical component of goal-directed actions, though some published findings are conflicting. We sought to reconcile the existing literature by comparing the effects of excitotoxic lesions of the perigenual anterior cingulate cortex (pgACC), a region of the mPFC, and OFC on contingency learning in the marmoset monkey using a touchscreen-based paradigm, in which the contingent relationship between one of a pair of actions and its outcome was degraded selectively. Both the pgACC and OFC lesion groups were insensitive to the contingency degradation, whereas the control group demonstrated selectively higher performance of the nondegraded action when compared with the degraded action. These findings suggest the pgACC and OFC are both necessary for normal contingency learning and therefore goal-directed behavior.

## Introduction

Optimal decision-making is important in the face of a dynamic and often unpredictable environment. Two separate learning processes are theorized to contribute to decision-making: a goal-directed mechanism whereby links are made between actions and their outcomes, and a habit-based system in which stimulus-response associations are formed (Dickinson 1985). Disruption in the balance between the two processes, and hence impaired decision-making, has been suggested to play a role in the pathology of a number of neuropsychiatric disorders including schizophrenia (Morris et al. 2015), addiction (Everitt et al. 2001; Everitt and Robbins 2005; Sjoerds et al. 2013; Everitt 2014), excessive and compulsive behaviors in eating disorders (Smith and Robbins 2013; Godier and Park 2014), depression (Griffiths et al. 2014), and obsessive-compulsive disorder (OCD) (Gillan et al. 2011).

Multiple variables are evaluated in the choice to make a goal-directed action. For example, the value of the potential outcome is weighed against the cost entailed by the action, as well as how likely it is that the proposed action will result in the desired outcome. However, this knowledge alone is insufficient to promote the execution of a particular action; the likelihood of the outcome occurring without the action being performed must also be taken into account (Schultz 2015). This dichotomy was first formalized in Pavlovian conditioning as the concept of contingency (Rescorla 1967, 1968), and can be defined in an instrumental context as the difference between the probability of reinforcer delivery given a response and the probability of reinforcer delivery in the absence of that response (Hammond 1980).

The subjective value of the outcome and the contingent relationship between the action and the outcome are thus both important facets of goal-directed actions. They can be assessed using outcome revaluation and contingency degradation tests, respectively, thereby determining whether behavior is goal-directed or habitual. In the former the value of the outcome is altered, for example by inducing sensory-specific satiety (Colwill and Rescorla 1985a; Rolls 1986; Hetherington and Rolls 1996; Balleine and Dickinson 1998a) or a conditioned aversion to a food reward (Adams and Dickinson 1981; Colwill and Rescorla 1985a, 1985b) or by changing the subject's motivational state (Dickinson and Dawson 1987; Dickinson 1989). A re-direction of instrumental behavior consistent with the new outcome value occurs if the behavior is goal-directed in nature but not if it is habitual (Adams 1982; Colwill 1993; Dickinson and Balleine 1995). Following contingency degradation, the subject of this article, the contingent relationship between the action and its outcome is weakened by the delivery of noncontingent outcomes. Performance of the action declines if it is goal-directed, an effect which has been shown in rats (Hammond 1980; Dickinson and Charnock 1985; Balleine and Dickinson 1998b), mice (Gourley et al. 2013a, 2013b) and humans (Chatlosh et al. 1985; Shanks and Dickinson 1991); a similar effect is also seen in human causal judgments (Allan and Jenkins 1980; Wasserman et al. 1983; Chatlosh et al. 1985; Neunaber and Wasserman 1986; Shanks and Dickinson 1988, 1991).

The medial prefrontal cortex (mPFC) of rats has been implicated in behavioral sensitivity to contingency degradation. A pre-training lesion of the rat prelimbic cortex (PL) has been shown to induce insensitivity to subsequent contingency degradation (Balleine and Dickinson 1998b). There is disagreement however regarding the sector of primate mPFC to which the rodent PL corresponds (Myers-Schulz and Koenigs 2012). Based on the findings from human neuroimaging studies of contingency learning (Tanaka et al. 2008; Liljeholm et al. 2011), it has been suggested that an anterior part of ventromedial PFC that encroaches on Brodmann area (BA) 10/14 may be equivalent to PL (Balleine and O'Doherty 2010). In contrast, PL has also been likened to dorsal anterior cingulate cortex (ACC), BA 24, in humans since both regions have been implicated in the regulation of conditioned fear (Milad and Quirk 2012). However, consideration of cytoarchitecture and receptor distribution points to primate perigenual anterior cingulate cortex (pgACC), area 32, as equivalent to PL (Gabbott et al. 2003; Vogt et al. 2013). These discrepancies highlight the need to perform experimental studies of contingency learning in a nonhuman primate species, in which the structure and functional organization of PFC has a greater similarity to humans compared with that of rodents (Uylings and van Eden 1991). We therefore chose to investigate the effects of selective excitotoxic lesions of pgACC (area 32) of the common marmoset, a New World monkey, on contingency learning.

The orbitofrontal cortex (OFC) is another key region thought to contribute to goal-directed behavior. Its role is considered to stem primarily from its involvement in outcome expectancy and consequent effects on choice (Holland and Gallagher 2004; Schoenbaum and Roesch 2005), but the outcome expectancies are usually thought to derive from Pavlovian stimuli- rather than action-outcome associations, evidence for which comes from studies looking at the effects of lesions to lateral regions of OFC in both rhesus monkeys (areas 11 and 13 based on Carmichael and Price 1995; Izquierdo et al. 2004; Machado and Bachevalier 2007; Baxter et al. 2009; Rudebeck and Murray 2011; West et al. 2011; Rhodes and Murray 2013) and rodents (Gallagher et al. 1999; Pickens et al. 2003, 2005). However, it should be noted that in the rhesus studies the stimuli were presented in an instrumental context making it difficult to rule out action-outcome associations contributing to the behavior. In addition, human functional neuroimaging has demonstrated modulation of medial OFC (area 14/10) activity during devalued but not valued action selection (Gottfried et al. 2003; Valentin et al. 2007). In the majority of these studies of OFC involvement in the associations between stimuli, actions and outcomes, behavioral tests have focused on outcome revaluation, rather than the contingent relationship between these variables. An exception is the work of Ostlund and Balleine (2007), who showed that large lesions of ventral and lateral OFC in rats disrupted behavioral sensitivity to the degradation of stimulus-outcome contingencies, though action-outcome contingency degradation was not studied. Increased activity during goal-directed, as opposed to habitual actions, has been reported in neurons of ventral and lateral OFC in mice and chemogenetic inactivation or optogenetic activation of the area decreased or increased, respectively, the level of goal-directed behavior (Gremel and Costa 2013). Moreover, a recent study has implicated the medial OFC of rats in using the knowledge of the relationship between actions and their outcomes to inform goal-directed behavior but only when the information is not present at the time of test (Bradfield et al. 2015). A test of contingency degradation, similar to that used in the present study, was not affected by medial OFC lesions (Bradfield et al. 2015), but see Gourley et al. (2010).

Given that the role of the primate OFC specifically in learning or using information about the contingent relationship between actions and their outcomes to guide choice is still unclear, the present study compared the role of primate pgACC (area 32) and lateral OFC (primarily areas 11, 13 according to the marmoset atlas of Paxinos et al. 2012) in contingency learning for action-outcome associations. The behavioral sensitivity to contingency degradation was assessed in marmosets using a computerized touchscreen version of a paradigm developed by Hammond (1980) and Balleine and Dickinson (1998b). Following pre-training excitotoxic lesions of pgACC or OFC, animals were trained on alternating sessions to respond to one of the two stimuli associated with two different rewards, presented on either side of the center of a touchscreen. Subsequently, their sensitivity to contingency degradation was investigated by reducing the contingent relationship between responding to one of the stimuli and its associated reward but not altering the contingent relationship between responding to the other stimulus and its associated reward.

## Materials and Methods

### Subjects

Fourteen common marmosets (*Callithrix jacchus*; 8 females, 6 males), bred on site in a conventional barrier facility at the University of Cambridge Marmoset Breeding Colony, were housed in pairs in purpose-built housing. Rooms were maintained at 24°C and 55% relative humidity and were gradually illuminated from 07.00 to 07.30 and dimmed from 19.00 to 19.30, following a 12 h light/dark cycle with dawn and dusk. Males had received a vasectomy to prevent any pregnancies in their female partners. On weekdays, all subjects were fed 20 g of MP.E1 primate diet (Special Diet Services, Essex, UK) and one piece of fresh fruit after the daily behavioral testing session, with simultaneous access to water for 2 h. On weekends, their diet was supplemented with marmoset jelly (Special Diet Services), peanuts, fresh fruit and eggs and access to water was ad libitum. All monkeys were regularly assessed by the Named Animal Care and Welfare Officer and the Named Veterinary Surgeon. Their cages contained a variety of environmental enrichment aids that were regularly varied. All procedures were performed in accordance with the UK Animals (Scientific Procedures) Act 1986 under project license 80/01344.

### Apparatus

Behavioral testing took place in an automated, sound-attenuated apparatus within a darkened room. Subjects sat in a transparent Perspex box within the apparatus, one side of which was removed to reveal a touch-sensitive screen (Intasolve). Subjects reached through an array of metal bars to manipulate the touchscreen, upon which computer-controlled stimuli, programmed in-house, were presented. A centrally placed licking spout, containing four tubes connected to separate pumps (Autoclude), allowed the delivery of up to four liquid reinforcers, though in this study only two reinforcers were used. Two tone generators (RS Components) were present on either side of the screen and the apparatus was lit by a 3W light bulb. All experiments were monitored with a video camera mounted from the roof of the apparatus.

### Surgical Procedures

**Figure 1.**
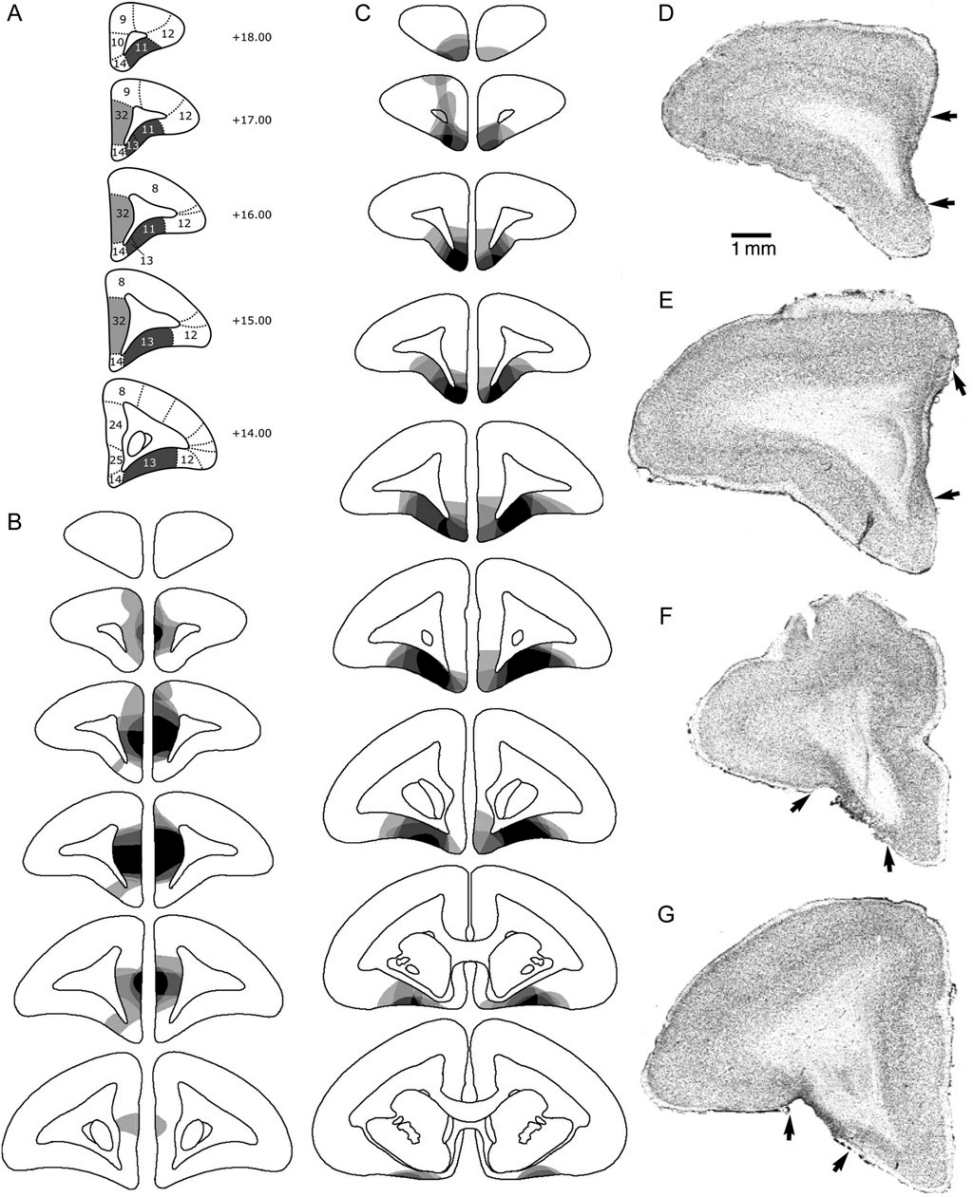
(*A*–*C*) Schematic diagrams of a series of coronal sections through the frontal lobe of the marmoset, illustrating the site of the lesion of the pgACC and OFC. (*A*) Diagram showing target regions for the pgACC (light gray) and OFC (dark gray). (*B*,*C*) The different levels of shading, ranging from solid black to pale gray, represent the areas of cortex that were damaged in all monkeys, in all monkeys but one, etc., to just one monkey, in pgACC and OFC, respectively. (*D*–*G*) Photomicrographs of cresyl fast violet-stained coronal sections through rostral (*D*,*F*) and intermediate (*E*,*G*) levels of the prefrontal cortex taken from a representative marmoset from the pgACC- (*D*,*E*) and OFC (*F*,*G*)-lesioned groups. The extensive cell loss in the lesioned areas is in stark contrast to the dense layering of neurons seen in the adjacent intact areas. In addition, the loss of orbitofrontal tissue in the OFC-lesioned monkey is in contrast to the intact OFC in the pgACC-lesioned monkey (*D*,*E*) and vice versa. The arrows mark the borders of the lesions. Cytoarchitectonic numbering according to (Paxinos et al. 2012).

Four monkeys received excitotoxic pgACC lesions, five received excitotoxic OFC lesions and five received a sham control procedure (Fig. 1). Excitotoxic lesions of the pgACC were made by infusing 0.3–0.7 µL/site of a 0.09 M solution of quinolinic acid (Sigma) in 0.01 M phosphate buffer, pH 7.0, bilaterally into three sites. Excitotoxic lesions of the OFC were made by infusing 0.4–0.6 µL/site of a 0.09 M solution of quinolinic acid (as described above) bilaterally into eight sites. Surgical co-ordinates are given in Table 1. Surgical procedures have previously been described in full (Pears et al. 2003).

**Table 1:** Stereotactic co-ordinates for pgACC and OFC lesions

AP (in mm)	LM (in mm)	Cannula position from skull base^a^ or brain surface^b^ (in mm)
Orbitofrontal cortex lesion		
+16.00	±2.0	0.7^a^
+16.00	±4.5	0.8^a^
+16.75	±2.5	0.7^a^
+16.75	±4.0	0.7^a^
+17.75	±2.0	0.7^a^
+17.75	±4.0	0.7^a^
+18.50	±2.0	0.7^a^
+18.50	±3.0	0.7^a^
Perigenual ACC lesion		
+16.75	±2.5	2.6^b^
+17.50	±1.0	2.6^b^
+18.50	±0.75	2.0^b^

AP, anteroposterior from the interaural line; LM, mediolateral from the midline.

### Preliminary Behavioral Training Procedures

**Figure 2.**
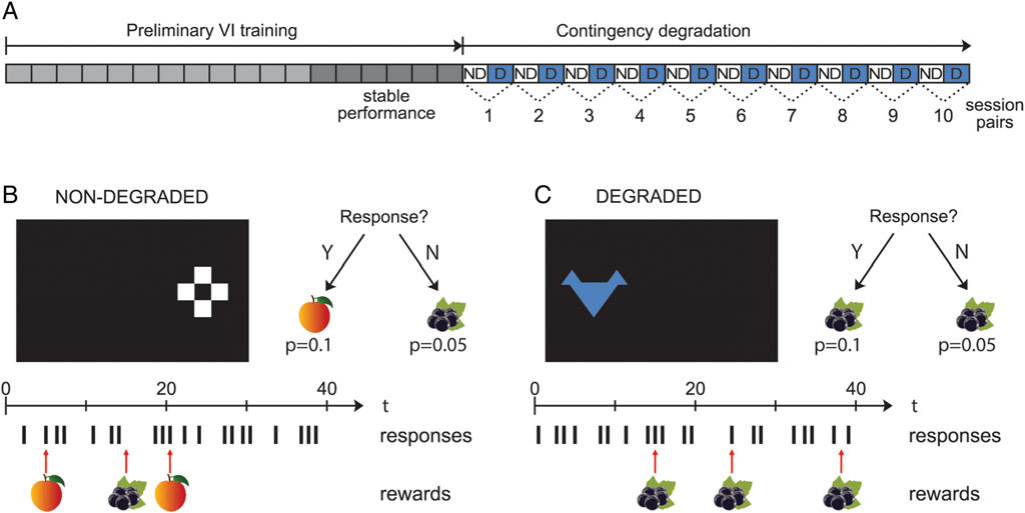
Example contingency degradation for the pairing of response to the left stimulus with blackcurrant juice. (*A*) Schematic diagram outlining the schedule of sessions in the training phase and the subsequent contingency degradation test. Following 18 sessions of preliminary VI training, subjects were presented with 20 sessions of the contingency degradation test. On alternate sessions, the white stimulus was presented on the right (responding on which was associated with peach juice) and the blue stimulus was presented on the left (responding on which was associated with blackcurrant juice), as indicated by the white and blue shading of the sessions. For half the subjects, blackcurrant juice was delivered noncontingently across all sessions, whereas peach juice was delivered noncontingently for the others. In the 10 “degraded” sessions, shown by “D” labeling, the juice delivered noncontingently was the same as that delivered contingent upon responding, and in the 10 “nondegraded” sessions, represented by “ND” labelling, the juice delivered noncontingently was not the same as that delivered contingent upon responding. In this example, the contingent relationship between responding to the left blue stimulus and delivery of blackcurrant juice is degraded. (*B*,*C*) Illustration of stimuli and their relative positions on the touchscreen along with a simulated series of responses with contingent and noncontingent rewards. (*B*) Nondegraded contingency condition. Subjects receive peach juice reward for responding to right stimulus with *P* = 0.1 and noncontingent blackcurrant juice reward with *P* = 0.05. (*C*) Degraded contingency condition. Subjects receive blackcurrant juice reward for responding to left stimulus with *P* = 0.1 and noncontingent blackcurrant juice reward with *P* = 0.05.

All subjects had previous experience of behavioral tests presented on a touchscreen (a series of visual discrimination tasks involving second-order schedules of reinforcement as previously described [Experiments 1, 2 and 4; Pears et al. 2003]). Following the conclusion of the experiments of Pears et al. (2003), subjects had a break from behavioral testing of several weeks' duration. Subjects were then pre-exposed to the two liquid reinforcers to be used, blackcurrant and peach 20% maltodextrin solutions, in the home cage. Subsequently, subjects were trained to perform two distinct actions to gain receipt of each reinforcer in the test apparatus. Touching a white stimulus on the right hand side of the screen resulted in delivery of peach juice, while touching a blue stimulus on the left resulted in delivery of blackcurrant juice (Fig. 2*B*,*C*). Actions were trained separately in 30 min alternating sessions and subjects had one session daily, 5 days a week.

Subjects were initially trained to perform the actions under a variable interval (VI) 3.5 s (range 2–5 s) schedule of reinforcement, whereby after a VI had elapsed the next response resulted in 10 s delivery of the associated juice through the licking spout, followed immediately by the commencement of the next VI. All responses were recorded and each response resulted in a 1 s disappearance of the stimulus from the screen. If the response was not rewarded, the stimulus re-appeared. For each action-outcome association, there were three sessions of VI 3.5, followed by three sessions of VI 10 (range 5–15 s) and finally three sessions of VI 20 (range 15–25 s), making a total of 18 sessions (Fig 2*A*). During the final six sessions, animals displayed stable levels of responding.

### Contingency Degradation

Following completion of the preliminary training procedures, the contingency was partially degraded for one of the response-outcome pairings, but not the other (Fig. 2). The same actions and outcomes were available as described in VI training, and the action-outcome pairings were still presented in separate, alternating sessions. A contingency degradation session was organized into a series of 1 s bins, where the probability of reinforcement given a response in each bin was 10% (P(O|A) = 0.1). However, there was also a 5% probability of the delivery of juice in every bin in which there was no response (P(O|∼A) = 0.05). For one of the actions, the juice that was available noncontingently was the same as the contingent juice (counterbalanced across subjects) and thus, for those sessions the action-outcome contingency was partially degraded (“degraded session”). For the other action, the juice that was available noncontingently was not the same as the contingent juice, and thus by continuing to respond the animal could gain access to two different juices (“nondegraded session”). Each of the action-outcome associations was presented in alternate sessions on 10 occasions making a total of 20 sessions. The action-outcome contingency that was degraded and the order of degraded versus nondegraded alternating sessions were counterbalanced across subjects.

### Behavioral Measures

Responding during the contingency degradation sessions was analyzed using two measures: 1) absolute levels of responding and 2) a ratio score, to control for differences in the absolute levels of responding across subjects. To calculate the ratio score, the 20 sessions of the contingency test were divided into 10 pairs of contiguous sessions (i.e., Sessions 1 and 2, Sessions 3 and 4 etc.), and thus each pair comprised one nondegraded session and one degraded session. For each pair, the number of responses in the nondegraded session was divided by the sum of responses in both the degraded and nondegraded conditions (i.e., nondegraded/(degraded + nondegraded)). Thus, the ratio score represents the number of responses in the nondegraded session as a proportion of the total responses made across both degraded and non-degraded sessions, with a value greater than 0.5 indicating a greater number of responses in the nondegraded condition relative to the degraded condition. A value of 0.5 would indicate an equal number of responses in both conditions.

### Statistical Analysis

All behavioral data were analyzed using SPSS Statistics v22 (IBM). Data were assessed using repeated-measures analysis of variance (rmANOVA). Appropriate transformations were used if data violated the assumptions of ANOVA, including the Huynh-Feldt correction when within-subject effects were found to be nonspherical. Post hoc pairwise comparisons were made between individual data points based on the estimated marginal means with the least squares difference (LSD) adjustment.

### Histological Procedures

All monkeys were perfused transcardially with 500 mL of 0.1 M PBS, pH 7.4, followed by 500 mL of 4% paraformaldehyde fixative administered over 10 min. The entire brain was removed and placed in a fixative solution overnight before being transferred to a 30% sucrose solution for a minimum of 48 h before sectioning. The sucrose solution served as a cryoprotectant during subsequent sectioning of the brains. Coronal sections were cut on a freezing sledge microtome at a thickness of 60 µm. Every third section was mounted on a gelatin-coated glass microscope slide and stained with cresyl fast violet. Sections were viewed under a microscope (Aristophot) and used to identify the lesioned area, which was defined by major neuronal loss often accompanied by marked gliosis. For each marmoset, the size and extent of the lesion was schematized onto drawings of a series of coronal sections through the marmoset PFC depicting every other section. Subsequently, these drawings were overlaid, and a composite figure was produced to illustrate the cortical area that was lesioned in all animals and the areas only lesioned in some of the animals. Photomicrographs of the pgACC and OFC at two different rostrocaudal levels within the PFC were taken at low Leitz Aristophot magnifications from representative lesioned subjects.

## Results

### Histological Analysis

Full details of the lesion have been described previously in Pears et al. (2003). In summary, the lesion of the pgACC (Fig. 1*D*,*E*) extended from just posterior of the frontal pole to just anterior of the head of the caudate nucleus (Fig. 1*B*). In the majority of cases, the damage was bilateral and in no cases did it extend into the ventromedial convexity (area 14) at the base of the brain. The lesion of the OFC (Fig. 1*F*,*G*) extended from the posterior edge of the frontal pole to just posterior to the genu of the corpus callosum (Fig. 1*C*). In most cases, it included the majority of the dysgranular regions (areas 11 and 13), sparing the more anterior granular regions. In four of the five animals, there was variable cell loss to the ventromedial convexity, greater anteriorly than posteriorly, and greater on the left than the right.

### Lack of Effect of Excitotoxic Lesions of pgACC or OFC on Acquisition of VI Responding

Animals in all three groups successfully completed VI training at similar performance levels. Analysis of response levels across the three VI20 sessions prior to contingency degradation confirmed that there were no differences between groups (analysis of variance [ANOVA]: *F* < 1). There was also no difference in performance between sessions using the two different stimuli (*F* < 1).

### Insensitivity to Contingency Degradation in pgACC and OFC Lesioned Groups

**Figure 3.**
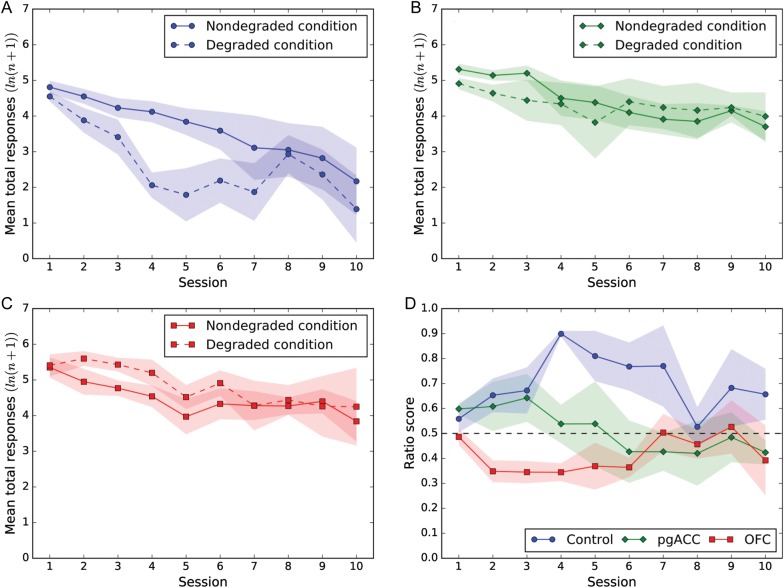
(*A*–*C*) Mean total numbers of responses (log transformed) across sessions for each group. Responding in the degraded (dotted line) and nondegraded (solid line) conditions are shown. (*A*) Control *n* = 5. (*B*) pgACC *n* = 4. (*C*) OFC *n* = 5. (*D*) Ratio scores showing mean responses normalized for the overall response rates of individual animals. The solid fill surrounding each point represents the standard error of the mean. The ratio score was calculated for each pair by dividing the number of responses in the nondegraded session by the sum of the responses from the degraded and nondegraded sessions (nondegraded/(nondegraded + degraded)). The ratio score therefore represents the proportion of responses in the nondegraded condition relative to the degraded condition with a value >0.5 indicating a greater number of responses in the nondegraded condition relative to the degraded condition.

Overall, responding declined over the course of the contingency degradation (Fig. 3), as revealed by a main effect of Session pair (*F*_4.44,48.79_ = 11.37; *P* < 0.001), but as predicted, responding in the control group declined more quickly in the partial contingency degradation condition compared with the nondegraded condition. This differential pattern of responding was not seen, however, in either the pgACC or OFC lesioned groups. Thus, overall both lesioned groups appeared insensitive to contingency degradation.

Total responses were transformed using the natural logarithm to achieve homogeneity of variance by Levene's test prior to analysis by rmANOVA with between-subject factors of Group (pgACC, OFC, sham) and within-subject factors of Contingency (degraded, nondegraded) and Session pair (1–10). As some sessions contained zero values of responses, the transformation ln(*x* + 1) was used. rmANOVA revealed a main effect of Group (*F*_2,11_ = 4.33, *P* < 0.05) such that control subjects responded significantly less across contingency degradation than the OFC-lesioned group (pairwise comparisons [LSD]: control versus OFC [*P* < 0.05]) with a trend level difference for the pgACC-lesioned group (*P* = 0.055). The two lesioned groups did not differ from one another (*P* = 0.64). Most importantly, there was a Group × Contingency interaction (*F*_2,11_ = 5.48, *P* < 0.05) which revealed that only the control group showed a greater reduction in responding in the degraded condition compared with the nondegraded condition (pairwise comparisons [LSD], *P* < 0.01). In contrast, the pgACC- and OFC-lesioned groups showed no significant difference between the two conditions (pgACC: *P* = 0.28, OFC: *P* = 0.24). While it may look like the OFC-lesioned group showed the opposite effect to controls in Figure 3*C*, namely more responding during the early sessions of degradation in the degraded compared with the nondegraded condition, this is not significant when analyzed by rmANOVA. Even if analysis is performed on the OFC group independently of the other Groups, there is no Session × Contingency interaction (*F* < 1).

There were no other main or interaction effects [Contingency (*F*_1,11_ = 3.67, *P* = 0.082); Session × Contingency (*F*_9,99_ = 1.32, *P* = 0.24); Session × Group (*F* < 1) although there was a strong trend toward a Session × Contingency × Group interaction (rmANOVA: *F*_18,99_ = 1.67; *P* = 0.057)]. Comparison of Figure 3*A*–*C* suggests that the latter interaction was the result of differential responding between degraded and nondegraded conditions developing across sessions in the control group but not in the lesioned groups.

To control for potential variation in response levels—which could obscure differences between groups—the ratio of nondegraded versus total responses was calculated for each subject (see Materials and Methods). Whereas the performance of animals in the control group showed a robust decline in responding following contingency degradation, as indicated by ratio scores well above 0.5, the performance of animals in the lesioned groups did not, their ratio scores remaining close to 0.5 throughout, as evidenced by a Group × Session interaction (*F*_18,99_ = 1.75, *P* < 0.05). The average ratio scores across all sessions were significantly different across groups (Fig. 3*D*; rmANOVA: *F*_2,11_ = 5.65, *P* < 0.05). Pairwise comparisons showed that the ratio scores across all sessions of the control group were higher than those of the OFC lesion group (*P* < 0.01) and the elevation of the control group ratio scores compared with those of the pgACC lesion group trended to significance (*P* = 0.063). Overall ratio scores did not differ between lesioned groups (*P* = 0.31).

## Discussion

Excitotoxic lesions of pgACC or OFC rendered marmosets insensitive to contingency degradation. Sham-operated control subjects reduced their responding in sessions in which the action-outcome contingency had been degraded compared with nondegraded contingency sessions. In contrast, subjects with pgACC or OFC lesions showed no differential responding, maintaining their performance of both actions regardless of whether one of the action-outcome contingencies had been degraded or not. Neither lesioned group, however, showed altered levels of responses during acquisition of the variable interval schedule of responding for juice reward. Together, these findings provide new insight into our understanding of the prefrontal contribution to the instrumental control of behavior.

The task design was an adaptation for marmosets of that used by Balleine and Dickinson (1998b), based on the original work of Hammond (1980) in rats. Hammond (1980) demonstrated that animals detect and use contingency information in instrumental responding. However, an alternative explanation of the Hammond result was that the delivery of noncontingent reward could strengthen competing responses, such as approach to the reward source, and thus it would be impossible to conclude that the reduction in responding was due specifically to sensitivity to contingency degradation (Colwill and Rescorla 1986; Dickinson and Mulatero 1989; Balleine and Dickinson 1998b). A subsequent modification of the design (Colwill and Rescorla 1986; Dickinson and Mulatero 1989; Williams 1989; Balleine and Dickinson 1998b) introduced a second action-outcome pairing which is not degraded during the experiment but nevertheless occurs alongside the delivery of noncontingent reward, a design feature also incorporated into the current study. Any response competition induced by the presence of the noncontingent reward would be expected to affect the nondegraded action-outcome pairing to the same extent as the degraded action-outcome pairing, and so any difference in levels of responding between the two must be intrinsic to the contingency degradation itself.

Sham-operated control subjects performed much as expected in the contingency degradation test, with significantly reduced responding in the degraded contingency sessions compared with the nondegraded contingency sessions (Fig. 3*A*), indicative of behavioral sensitivity to alterations in contingency. However, they also showed a generalized reduction in responding across all sessions of the contingency test, which was not anticipated. One likely cause of this generalized reduction is a gradual decrement of motivation to respond due to the cumulative effect of free reward delivery across multiple sessions. The effect, however, was sufficiently retarded to allow the differential responding to the degraded and nondegraded sessions to be revealed in the middle phases of the contingency test. A similar gradual decline in responding across sessions was also present in the pgACC- and OFC-lesioned groups.

A considerable body of work implicates mPFC, and more specifically the PL region of the rat, in the encoding of action-outcome associations. Excitotoxic lesions of this region disrupt sensitivity to both contingency degradation (Balleine and Dickinson 1998b) and to outcome devaluation (Corbit and Balleine 2003; Killcross and Coutureau 2003; Coutureau et al. 2009), with the former also being impaired by dopamine depletion (Naneix et al. 2009), but see Lex and Hauber (2010). In addition, selective knockdown of *brain-derived neurotrophic factor* in PL cortex increases sensitivity to contingency degradation (Hinton et al. 2014) while exposure to chronic stress, which has long been known to induce atrophy of mPFC (Radley et al. 2004; Cerqueira et al. 2007), including PL cortex, gives rise to insensitivity to both outcome devaluation and contingency degradation (Dias-Ferreira et al. 2009). Moreover, the role of PL cortex in action-outcome learning has been further specified by studies showing that lesions or transient inactivations of PL cortex 1) disrupt the acquisition but not the expression of action-outcome associations (Ostlund and Balleine 2005; Tran-Tu-Yen et al. 2009) and 2) resolve conflict between action-outcome and stimulus-response representations in the control of behavioral output by reducing the influence of the former (Dwyer et al. 2010).

In primates, the more dorsal aspects of ACC, particularly those regions lying in and around the cingulate sulcus (primarily area 24 but excluding perigenual area 32), have been implicated in action-outcome learning. Human neuroimaging shows activation in the dorsal ACC when choices should be changed in response to reduced reward value (Bush et al. 2002), and neurons in the dorsal ACC of macaques appear to encode particular action-outcome associations (Matsumoto et al. 2003). The deficits in action-based reversal learning induced by ablations of the sulcal regions of ACC have also been interpreted as impairments in the encoding of action-outcome relationships (Shima and Tanji 1998; Kennerley et al. 2006; Rushworth et al. 2011). In addition, the finding that lesions of the ACC in macaques impair performance of a reward-conditional response selection task but not a visual discrimination task, was interpreted as evidence of a selective role for the ACC in responding under the control of action—but not stimulus—outcome associations (Hadland et al. 2003). Only in the latter study did the damage extend into the more anterior and ventral regions of perigenual area 32, but whether damage to area 32, or damage to the more dorsal area 24, was responsible for the deficit could not be determined. More recently, findings that both stimulus- and action-based reversal learning are impaired by lesions of macaque dorsal ACC (Chudasama et al. 2013), again excluding area 32, have prompted the suggestion that the region may play a more general role in rewarded behavior. The only study to have focused specifically on the effect of lesions of primate area 32 on action-outcome learning showed equivocal effects on behavioral choice following the devaluation of outcomes (Rhodes and Murray 2013). However, until now, the specific contribution of perigenual area 32 to the encoding of the contingent relationship between action and outcomes has not been studied. Moreover, it should be noted that with respect to both area 32 and dorsal area 24, the use of ablations in macaques makes it impossible to rule out damage to fibers of passage underlying any observed deficits in those investigations.

The present study used localized excitotoxic lesions to investigate the specific contribution of the ACC to action-outcome learning, focusing on area 32 within the pgACC, as it has the greatest structural similarity to the rodent PL (Gabbott et al. 2003; Vogt et al. 2013). The lesions spare not only neighboring area 24, but also any fibers of passage. The finding that excitotoxic lesions of area 32 led to insensitivity to contingency degradation demonstrates the critical contribution of area 32 to goal-directed actions. It is consistent with the over-responding these same animals showed on a progressive ratio schedule for primary food reward, and their retarded return to baseline levels of responding after the reintroduction of reward following a reward omission test (Pears et al. 2003). Whether the disruption in contingency sensitivity is due to a loss of sensitivity to stimulus- or action-outcome contingencies, however, cannot be determined in the present study. Each of the two rewards was paired with both distinct stimuli and distinct actions (white stimulus on the right, blue stimulus on the left) and thus it is unclear which association controlled responding. However, it should be noted that PL lesions in rodents also disrupt responding in Pavlovian-conditioned fear paradigms (Corcoran and Quirk 2007; Sharpe and Killcross 2015) and so it is likely that PL plays a more general or executive role, not specific to the learning of action-outcome associations. For example, it has recently been hypothesized that PL may play a role in attentional selection (Sharpe and Killcross 2015) governed by a variety of high-order predictive cues which may account for the range of deficits that occur following PL lesions, including those of attentional set shifting (Birrell and Brown 2000), action-outcome learning (Balleine and Dickinson 1998b) and conditioned fear (Corcoran and Quirk 2007). Whether such a hypothesis could apply to the functions of primate area 32 depends on whether future studies establish the functional equivalence of these two regions across species.

The effects of OFC lesions on contingency degradation have not previously been studied in macaques. However, large OFC ablations do impair performance on other behavioral tasks involving changes in contingencies between stimuli and actions and their outcomes, including probabilistic discriminations and instrumental extinction. For example, lesions of area 11 and 13 impair performance on a probabilistic discrimination task (three-armed bandit) in which the contingencies between stimuli and outcomes change across time, with a pattern of responding consistent with a deficit in representing the specific link between particular stimuli and their outcomes (Walton et al. 2010). Indeed, a deficit in the ability to retrieve information about the specific outcomes of stimuli was proposed to explain the failure of these same OFC-lesioned animals used in the present study to acquire a new response for a conditioned reinforcer (Burke et al. 2008), a result originally reported in Pears et al. (2003). OFC ablations have also been shown to impair instrumental extinction (Butter et al. 1963; Butter 1969; Izquierdo and Murray 2005), involving the complete omission of the outcome. This contrasts with contingency degradation, as used in the present study, which similarly involves the breaking of the link between action and outcome but with continued outcome delivery, thus lessening emotional effects of frustrative nonreward (Amsel 1958, 1992) that occur following complete reward omission. Contingency degradation can thus be considered to be an analogous process to extinction and is thought to depend upon the same associative changes (Rescorla 2001), while allowing a more accurate assessment of the effects of interrupting the action-outcome association (Rescorla and Skucy 1969). Recently more selective excitotoxic lesions within the OFC of macaques, specifically medial area 14, but not lateral area 11/13, produced impairments in extinction, which doubly dissociated with the effects of area 11/13 (but not area 14) lesions on reinforcer devaluation (Rudebeck and Murray 2011).

Given that the OFC lesion in marmosets in the present study extended into medial area 14, the contingency degradation deficit in our OFC group is consistent with the macaque area 14 lesion-induced extinction deficit (Rudebeck and Murray 2011), while ruling out alterations in frustrative nonreward as an alternative explanation. However, if area 14 were implicated, then the present results would be inconsistent with regard to the results of Bradfield et al. (2015), which showed that lesions of rat medial OFC disrupted retrieval of action-associated specific outcome representations only in situations when that information was not observable at the time of test; thus no deficits were seen during contingency degradation, a paradigm in which outcome information is observable. Rat medial OFC and primate BA 14 are not equivalent regions or alternatively, it may be that stimulus-outcome representations were driving instrumental responding in the marmosets as a consequence of the damage within lateral OFC (BA 11 and 13). If the latter, given that the outcomes were still observable at the time of test, it highlights the role of the lateral OFC in more than just retrieval of stimulus-associated specific outcome information.

Taken together, the results of the present study highlight how habit-like instrumental responding, whether driven by deficits in the retrieval of stimulus-outcome or action-outcome representations, is induced by damage to at least two distinct regions of PFC, namely OFC and pgACC. This has implications for our understanding of the neuropathological basis of disorders such as OCD and depression. For example, in the habit hypothesis of OCD (Graybiel and Rauch 2000; Gillan and Robbins 2014), it is thought that deficits in the regulation of goal-directed actions give rise to over-dominance of the habit system, and therefore to the intrusive, repetitive thoughts, and behaviors that are characteristic of OCD (Gillan et al. 2011, 2014c). Furthermore, there is evidence to suggest that altered contingency learning may be the cause of the goal-directed action deficits (Reuven-Magril et al. 2008; Gillan et al. 2014b), supporting the theory that OCD patients have a reduced sense of control over life events, and use compulsive behaviors to compensate, thereby imparting an illusory sense of control (Frost et al. 1993; McLaren and Crowe 2003; Moulding and Kyrios 2006; Moulding et al. 2007; Reuven-Magril et al. 2008; Gillan et al. 2014b). Recent work has linked the excessive habit formation seen in OCD with hyperactivation (Gillan et al. 2014a) and with reduced gray matter volumes (Voon et al. 2014) in OFC, a region known to be a key part of the fronto-striatal circuitry underlying both individual differences in the balance between goal-directed actions and habits (Gremel and Costa 2013) and in the pathophysiology of OCD (Evans et al. 2004; Menzies et al. 2008; Milad and Rauch 2012; Haber and Heilbronner 2013). Similarly, the theory of learned helplessness proposes that the phenomenon whereby exposure to uncontrollable aversive outcomes reduces the likelihood of subjects attempting to avoid such outcomes subsequently (Overmier and Seligman 1967; Seligman and Maier 1967; Hiroto 1974; Hiroto and Seligman 1975), may account for the symptomatology of depression (Seligman 1975; Rosenhan and Seligman 1984; LoLordo 2001). Uncontrollability describes a zero contingency condition between a subject's actions and the outcome (Seligman et al. 1971), a state which is proposed to induce an impairment in a subject's ability to perceive future contingent relationships (Maier and Seligman 1976; Abramson et al. 1978). It has recently been associated with damage to regions of the mPFC (Amat et al. 2005; Wang et al. 2014).

Together, the present findings have provided strong evidence of a role for both the pgACC and OFC of the marmoset monkey in instrumental responding but this now has to be considered in the broader context of more clearly defined fronto-striatal circuitry and its chemical neuromodulation, before the findings can be related with confidence to analogous investigations of contingency degradation in human patients.

## Funding

This research was supported by a Programme Grant (G0901884) from the Medical Research Council UK (MRC) to A.C.R., and a Wellcome Trust Senior Investigator Award (104631/Z/14/Z) to T.W.R. S.A.W.J. was supported by a BCNI-MRC studentship. The research was conducted at the Behavioural and Clinical Neuroscience Institute, which is supported by a joint award from the MRC and Wellcome Trust (G00001354). Funding to pay the Open Access publication charges for this article was provided by the Wellcome Trust and the MRC.
